# Threshold growth has a limited role in differentiating hepatocellular carcinoma from other focal hepatic lesions

**DOI:** 10.1186/s12880-023-01161-9

**Published:** 2023-12-04

**Authors:** Rong Lyu, Di Wang, Weijuan Hu, Zhongsong Gao, Changlu Yu, Jiao Wang, Mingge Li, Kefeng Jia

**Affiliations:** Department of Radiology, Tianjin Third Central Hospital, Tianjin Key Laboratory of Extracorporeal Life Support for Critical Diseases, Artificial Cell Engineering Technology Research Center, Tianjin Institute of Hepatobiliary Disease, NO.83 Jintang Road, Hedong District, Tianjin, 300170 China

**Keywords:** Hepatocellular carcinoma (HCC), Magnetic resonance imaging (MRI), Liver, Diagnosis

## Abstract

**Background and Objective:**

The role of threshold growth, as one of the major features (MFs) of hepatocellular carcinoma (HCC) in the Liver Imaging Reporting and Data System (LI-RADS) is inconsistent. This study evaluated the LI-RADS diagnostic performance for HCC when threshold growth was removed or replaced by independently significant ancillary features (AFs).

**Materials and Methods:**

This retrospective institutional review board-approved study included patients with a high HCC risk who underwent gadoxetic acid-enhanced MRIs. The MRI findings were consistent with pathologically proven focal hepatic observations. The pathological results were used as the gold standard reference. The sizes of the lesions with and without threshold growth were compared. Univariate and multivariate logistic regression analyses were used to confirm the independently significant AFs of HCC. In addition to the classification criteria of LI-RADS version 2018 (LI-RADS v2018), the lesions were also reclassified according to the following two schemes: scheme A, using all MFs except threshold growth, with threshold growth feature treated as an AF favouring malignancy; and scheme B, replacing the threshold growth feature with independently significant AFs and treated them as new MFs. The diagnostic performance of the above two LI-RADS schemes for HCC was calculated and compared with that of LI-RADS v2018.

**Results:**

A total of 379 patients and 426 observations were included. Threshold growth was not an independent significant MF for HCC diagnosis [odds ratio (OR), 1.0; 95% confidence interval (CI), 0.6–1.8; *p* = 0.927]. For all three groups of observations (HCCs, non-HCC malignancies, and benign lesions), the mean size with threshold growth was smaller than that without threshold growth (all *p* < 0.05). The nodule-in-nodule feature was an independent significant AF (OR, 9.8; 95% CI, 1.2–79.3; *p* = 0.032) and was used to replace threshold growth as a new MF in scheme B. The sensitivities of schemes A and B were 74.0% and 75.6%, respectively. The specificities of schemes A and B were the same (88.6%). None of the diagnostic performance metrics for HCC (sensitivity, specificity, accuracy) of either scheme A or B was significantly different from those of LI-RADS v2018 (all *p* > 0.05).

**Conclusion:**

Threshold growth is not an independently significant MF for HCC diagnosis. The diagnostic performance of LI-RADS for HCC is not affected regardless of whether threshold growth is removed from the list of MFs or replaced with an independently significant and more HCC-specific AF, which is the nodule-in-nodule feature.

## Introduction

With stringent criteria, the accurate and reliable noninvasive diagnosis of hepatocellular carcinoma (HCC) can be made with imaging examinations, such as computed tomography (CT) and magnetic resonance imaging (MRI) [1]. Thus there is no more need for further invasive pathological biopsies. Therefore, imaging studies play a crucial role in the diagnosis of HCC and can be used to guide subsequent treatments [2]. In particular, gadoxetic acid-enhanced MRI (EOB-MRI) can improve the detection rate and diagnostic accuracy for HCC [1].

The Liver Imaging Reporting and Data System (LI-RADS) published by the American College of Radiology (ACR) is used to assign a category to focal hepatic observations obtained for high-risk HCC patients [3]. The LI-RADS categories include LR-1 (definitely benign), LR-2 (probably benign), LR-3 (intermediate probability of malignancy), LR-4 (probably HCC), LR-5 (definitely HCC), LR-M (probably and definitely malignant but not HCC specific), and LR-TIV (definitely tumour-in-vein) [3]. Each category reflects a relative probability of benignity, malignancy or HCC in general. LI-RADS aims to standardize imaging data collection and reporting for HCC to enhance communication, reduce interobserver variability, promote quality assurance and improve diagnostic performance [4].

According to the CT/MRI LI-RADS, it describes many major features (MFs) and ancillary features (AFs) of hepatic imaging findings. The MFs of HCC include arterial-phase hyperenhancement (APHE), nonperipheral washout, enhancing capsule and threshold growth in addition to size. The LI-RADS algorithm uses a combination of MFs to assign initial categories and then adjusts the categories according to AFs [3].

Among the MFs, the contribution of threshold growth feature to the diagnosis is the most controversial. Chernyak et al. [5] suggested that removing threshold growth as an MF would cause a nonnegligible proportion of LR-5 observations to be downgraded to LR-4. However, Park et al. [6] reported that threshold growth was not a significant diagnostic indicator of HCC. In particular, LI-RADS version 2018 (LI-RADS v2018) simplified the definition of threshold growth to “size increase of a mass by ≥ 50% in ≤ 6 months”, which is based only on expert consensus and consistent with the rules of the Organ Procurement and Transplantation Network (OPTN) in the United States [7]. Therefore, threshold growth as an MF of HCC lacks strong support, particularly since non-HCC malignancies and some benign lesions, such as abscesses, also meet the threshold growth defined by LI-RADS. Thus its value is limited in distinguishing HCCs from other suspicious lesions. Therefore, further research is needed to study whether the removal of the threshold growth from the LI-RADS MFs would affect the HCC diagnosis. It is also critical to study whether there are some AFs that could replace the threshold growth for HCC diagnosis. In this paper, the above contents were studied.

## Materials and methods

### Study population

**Fig. 1:**
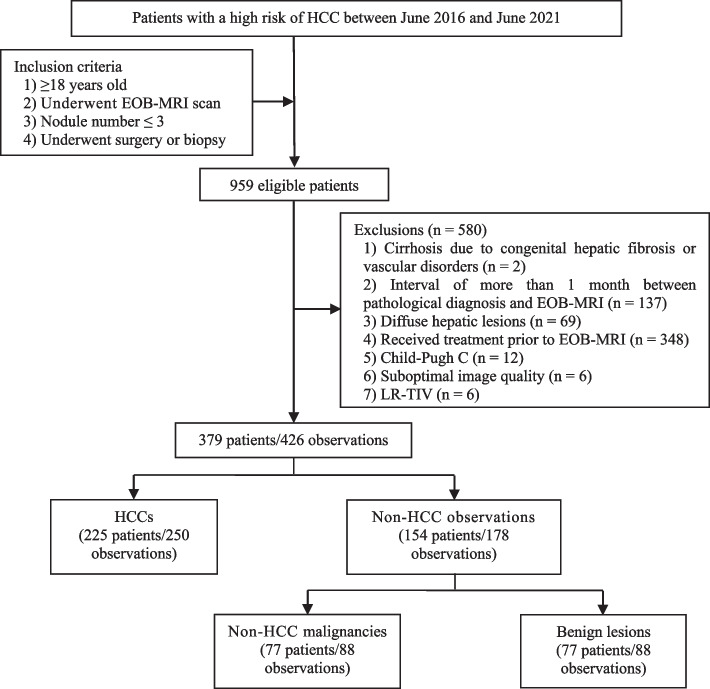
Flowchart of patient and observation selection. *HCC*, hepatocellular carcinoma; *EOB-MRI*, gadoxetic acid-enhanced magnetic resonance imaging; *LR (LI-RADS)*,Liver Imaging Reporting and Data System; *TIV*, tumour-in-vein

This study was approved by the Ethics Committee of Tianjin Third Central Hospital. Informed consent was waived due to the retrospective study design. Using electronic medical records, data from patients with a high risk of HCC [8] between June 2016 and June 2021 were retrospectively collected. The inclusion criteria were as follows: 1) ≥ 18 years old; 2) underwent EOB-MRI scans; 3) nodule number ≤ 3;and 4) underwent surgery or pathological biopsy. Patients meeting the following criteria were excluded from the study: (1) patients who had cirrhosis due to congenital hepatic fibrosis or vascular disorder; (2) interval of more than 1 month between pathological diagnosis and EOB-MRI; (3) diffuse hepatic lesions; (4) treatment prior to the EOB-MRI scans; (5) liver function was Child-Pugh C; (6) suboptimal image quality; or (7) LR-TIV (Fig. 1).

Patient clinicopathologic characteristics, including the age, sex, number of patients with cirrhosis, and the causes of liver disease were recorded through the patient records system. A board-certified radiologist with 4 years (J.W.) of experience in abdominal MRI reviewed all images and recorded the number of lesions.

### MRI techniques

For all examinations, studies were carried out by using a 3.0T MR system (Magnetom Verio, Siemens Healthcare, Erlangen, Germany) and an 8-channel phased-array torso coil. The liver MR imaging protocol consisted of in- and out-of-phase T1-weighted imaging (T1WI) acquired with a gradient recalled echo (GRE) sequence, respiratory-triggered axial T2-weighted turbo spin echo (TSE) sequence with fat suppression, free-breathing single-shot echo-planar diffusion-weighted imaging (DWI) with b values of 0, 50, 600, and 1000s/mm^2^. The liver MR imaging protocol also includes pre- and postcontrast T1-weighted three-dimensional volumetric interpolated breath-hold examination (VIBE) sequences acquired with a GRE sequence in the late arterial phase (AP) (30–35 s after aortic enhancement using the bolus tracking method), portal venous phase (PVP) (46–60 s), transitional phase (TP) (150–180 s), and hepatobiliary phase (HBP) (20 min after the contrast injection). Contrast-enhanced dynamic MR images of the liver were obtained after intravenous administration of gadoxetic acid (Primovist; Bayer Healthcare, Berlin, Germany) at 0.025 mmol/kg of body weight and a rate of 1.0 mL/s via a power injector, followed by 25 mL of 0.9% saline chaser at the same rate. The detailed parameters of each acquisition sequence are shown in Table 1.

**Table 1:** MR imaging protocol

Sequence	TR (ms)	TE (ms)	FA	ST (mm)	SS (mm)	FOV (mm^2^)	Scan matrix
In-phase T1WI	250	2.5	65°	6	1.5	380 × 285	512 × 300
Out-of-phase T1WI	250	3.8	60°	6	1.5	380 × 285	512 × 300
T2WI	3000–4000	91	90°	6	1.5	380 × 285	320 × 180
DWI	6000–7000	68	90°	6	1.5	400 × 300	128 × 78
VIBE	4.19	1.47	9°	4	0	400 × 300	320 × 168

*TR *Repetition time, *TE *Echo time, *FA *Flip angle, *ST *Slice thickness, *SS *Slice spacing, *FOV *Field of view, *T1WI *T1-weighted imaging, *T2WI *T2-weighted imaging, *DWI *Diffusion-weighted imaging, *VIBE *Volumetric interpolated breath-hold examination

### MRI analysis

All MRI scans were independently reviewed by two board-certified radiologists with 10 years (W.J.H.) and 6 years (D.W.) of experience in abdominal MRI, respectively. All MFs and AFs for each liver observation according to the LI-RADS v2018 were reviewed. All readers were blinded to the pathologic results. Discrepancies between the two readers were resolved by a third radiologist (R.L. with 17 years of experience in abdominal MRI) to reach a final consensus reading. For observations with documented threshold growth, readers determined the qualifying threshold growth criterion (≥ 50% diameter increase in ≤ 6 months) by retrospectively reviewing, measuring and comparing the observation size between current and prior exams results, with prerequisite that no treatment is performed between the two exams. The imaging exams, including EOB-MRI, contrast-enhanced MRI or CT (CE-MRI or CECT). The TP or HBP image from EOB-MRI and the PVP or delayed phase (DP) image from CE-MRI or CECT were selected to measure the observation size (the longest diameter on the plane with the largest cross-sectional area of the lesion), and the final lesion size was the average of the measurement results of 2 readers.

### Comparison of features and size of observations

Logistic regression analyses were performed to identify independent significant AFs for the diagnosis of HCC, which were then used as new MFs to replace the threshold growth feature. The sizes of observations with and without threshold growth were compared in the HCC, non-HCC malignancy and benign lesion groups.

### Category assignment and comparison of diagnostic performance

Every observation was initially assigned to a LI-RADS category according to the LI-RADS v2018 algorithm (first classified according to the MFs, then adjusted according to the AFs as follows: for ≥ 1 AF favouring malignancy, the LI-RADS category was upgraded by 1 up to LR-4; for ≥ 1 AF favouring benignity, the LI-RADS category was downgraded by 1; and for ≥ 1 AF favouring malignancy and ≥ 1 AF favouring benignity, the LI-RADS category remained unchanged).

Then, every observation was reassigned a category according to the following two schemes: Scheme A: the categories were assigned using all MFs except threshold growth, with only the threshold growth feature downgraded and treated as an AF favouring malignancy (not HCC in particular). Scheme B: the threshold growth feature was replaced with independently significant AFs and the latter were upgraded and treated as new MFs. LR-5 was considered as 100% definite HCC by LI-RADS classification, so the pathological results of LR-5 were taken as the gold standard reference. The diagnostic performance of schemes A and B for HCC diagnosis was calculated and compared with that of the unaltered LI-RADS v2018.

### Statistical analysis

Categorical variables were summarized as counts and percentages. Continuous variables were summarized as the means and standard deviations and were compared using Student’s *t-*test. The median (interquartile range) was used for nonnormally distributed data. To determine the AFs performance of HCC prediction, univariate and multivariate logistic regression analyses were performed. Variables with a *p* value < 0.1 in the univariable analysis were entered into the multivariable analysis to identify the independently significant AFs for HCC diagnosis. For the multivariable analysis, a stepwise backwards elimination method was used. Diagnostic performance was reported as the sensitivity, specificity, positive predictive value (PPV), negative predictive value (NPV), accuracy, and Youden index, and comparisons between grading protocols were made using *McNemar’s* test. Unless otherwise indicated, all statistical tests were two-tailed and conducted at the statistically significant level of 0.05 with *p* values reported. Statistical analyses were performed using SPSS Statistics version 25.0 (IBMCorp, Armonk, NY, USA).

## Results

### Patient characteristics and pathologic findings

The study population included 379 patients (265 males and 114 females; mean age, 58 ± 10 years) with 426 observations; of these, 314 (82.8%) patients had cirrhosis (diagnosed by typical imaging findings or biopsy). The causes of liver disease were hepatitis B virus (301 patients), hepatitis C virus (38 patients), alcohol consumption (9 patients), autoimmune disorders (4 patients), nonalcoholic steatohepatitis (1 patient), both hepatitis B and C virus (6 patients) and cirrhosis of unknown cause (20 patients). A total of 341 patients had a single observation, 29 patients had two observations, and 9 patients had three observations. Among the 426 observations (median size, 27 mm), there were 250 HCCs, 88 non-HCC malignancies and 88 benign lesions. Surgeries were performed for a total of 199 (46.7%) lesions (113 HCCs, 52 non-HCC malignancies, 34 benign lesions), and there were 227 (53.3%) lesions confirmed by biopsy (137 HCCs, 36 non-HCC malignancies, 54 benign lesions). The clinicopathologic characteristics of the patients and hepatic observations are shown in Table 2.

**Table 2:** Clinicopathologic characteristics of patients and hepatic observations

Characteristic	Value (%)
Patient (*n* = 379)	
Mean age (years)^a^	58.1 ± 10.4
Sex, male/female	265/114
Cirrhosis (%)	314 (82.8%)
Cause of liver disease (%)	
Hepatitis B virus	301 (79.4)
Hepatitis C virus	38 (10.0)
Alcohol consumption	9 (2.4)
Autoimmune disorders	4 (1.1)
NASH	1 (0.3)
Hepatitis B and C virus	6 (1.6)
Cirrhosis of unknown cause	20 (5.3)
Number of observations per patient (%)	
1	341 (90.0)
2	29 (7.7)
3	9 (2.4)
Observation (*n* = 426)	
Size (mm)	
Over all^b^	27 (16–52)
HCC^a^	34.7 ± 27.3
Non-HCC malignancy^b^	45 (27–66)
Benign lesion^b^	22 (14–44)
Final diagnosis (%)	
HCC	250 (58.7)
Non-HCC malignancy	88 (20.7)
ICC	41 (9.6)
CHC	13 (3.1)
Metastasis	23 (5.4)
Sarcomatoid carcinoma	6 (1.4)
Cystadenocarcinoma	3 (0.7)
Neuroendocrine carcinoma	1 (0.2)
Haemangiosarcoma	1 (0.2)
Benign lesion	88 (20.7)
Haemangioma	16 (3.8)
DN	34 (8.0)
RN	11 (2.6)
FNH	9 (2.1)
Adenoma or adenomatoid hyperplasia	6 (1.4)
Abscess	4 (0.9)
Angiomyolipoma	2 (0.5)
Epithelioid angiomyolipoma	3 (0.7)
Inflammatory nodule	1 (0.2)
Lipomyoma	1(0.2)
Ematoma	1 (0.2)
Standard reference of diagnosis (%)	
Surgical pathology	199 (46.7)
Biopsy pathology	227 (53.3)

Unless stated otherwise, data are presented as the number of patients or observations with the percentage in parentheses. ^a^Data are presented as the mean ± standard deviation; ^b^Data are presented as the median (interquartile range). NASHNonalcoholic steatohepatitis, *HCC* Hepatocellular carcinoma, *ICC *Intrahepatic cholangiocarcinoma, *CHC *Combined hepatocellular cholangiocarcinoma, *DN *Dysplastic nodule, *RN *Regenerative nodule, *FNH *Focal nodular hyperplasia

### Independently significant imaging features

**Fig. 2:**
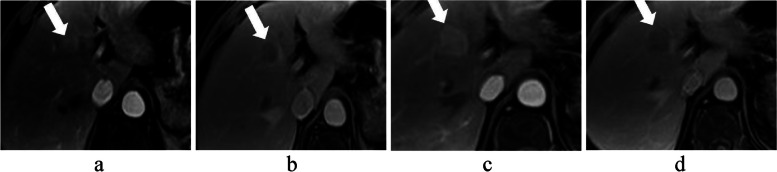
A 54-year-old man with HCC (biopsy pathology). EOB-MRI performed on July 25, 2017 demonstrated an 18-mm observation (arrow) in S4 of the liver with nonrim APHE (**a**) and washout on PVP (**b**). Then, EOB-MRI performed on September 5, 2017 (less than 2 months after the prior exam) showed that the nodule had grown to 29 mm with nonrim APHE (**c**), washout and an enhancing capsule on the PVP (**d**)

**Fig. 3:**
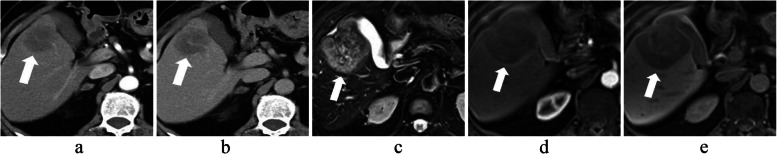
A 64-year-old man with liver metastases of rectal cancer (biopsy pathology). Liver CECT was performed on May 13, 2021. A 38-mm moderate inhomogeneous enhancement observation (arrow) was present in the arterial phase (**a**) and PVP (**b**). Then, EOB-MRI performed on November 3, 2021 (less than 6 months after the prior exam) showed that the lesion size had grown to 72 mm with moderate T2 hyperintensity on T2WI (**c**), no obvious arterial phase enhancement (**d**), and central enhancement in the PVP (**e**)

Among the MFs in LI-RADS, APHE [odds ratio (OR), 3.1; 95% confidence interval (CI), 1.7–5.7; *p* < 0.001], washout (OR, 10.7; 95% CI, 6.3–18.3; *p* < 0.001), and enhancing capsule (OR, 7.1; 95% CI, 3.7–13.6; *p* < 0.001) were independently significant imaging features of HCC diagnosis. Sixty-five of 250 HCCs and 69 of 176 non-HCC lesions (37 non-HCC malignancies and 32 benign lesions) among patients who had undergone previous imaging examinations (36 EOB-MRI, 20 CE-MRI, 78 CECT) within 6 months without treatment were used to determine the change in size. Among those observations, threshold growth was observed in 32 HCCs (Fig. 2) and 22 non-HCC lesions (Fig. 3). Threshold growth was not an independent significant MF for HCC (OR, 1.0; 95% CI, 0.6–1.8; *p* = 0.927). Among the AFs, multivariate logistic regression analyses showed that nodule-in-nodule appearance (OR, 9.8; 95% CI, 1.2–79.3; *p* = 0.032) was an independently significant imaging feature for HCC (Table 3).

**Table 3:** Logistic regression analysis of features for diagnosing HCC

	HCC (*n* = 250)	Non-HCC (*n* = 176)	Univariable analysis		Multivariable analysis	
			OR	*p* value	OR	*p* value
Major features						
APHE	220 (88.0)	95 (54.0)	6.25 (3.86–10.14)	< 0.001	3.12 (1.71–5.70)	< 0.001
Washout	193 (77.2)	31 (17.6)	14.73 (9.08–23.92)	< 0.001	10.74 (6.30-18.28)	< 0.001
Enhancing capsule	117 (46.8)	17 (9.7)	8.23 (4.71–14.38)	< 0.001	7.13 (3.74–13.61)	< 0.001
Threshold growth^a^	32 (12.8)	22 (12.5)	1.03 (0.58–1.84)	0.927		
Ancillary features						
Subthreshold growth^b^	37 (14.8)	15 (8.5)	1.87 (0.99–3.51)	0.054		
Corona enhancement	17 (6.8)	12 (6.8)	0.99 (0.46–2.14)	0.994		
Fat sparing in solid mass	4 (1.6)	2 (1.1)	1.42 (0.26–7.81)	0.691		
Restricted diffusion	229 (91.6)	147 (83.5)	2.15 (1.18–3.91)	0.012		
Mild-moderate T2 hyperintensity	232 (92.8)	143 (8.1)	3.62 (1.90–6.89)	< 0.001		
Iron sparing in solid mass	0 (0)	0 (0)	-	-		
TP hypointensity	222 (88.8)	122 (69.3)	3.51 (2.11–5.83)	< 0.001		
HBP hypointensity	236 (94.4)	142 (80.7)	4.04 (2.10–7.78)	< 0.001		
No-nenhancing capsule	8 (3.2)	3 (1.7)	1.91 (0.50–7.29)	0.346		
Nodule-in-nodule	15 (6.0)	1 (0.5)	11.17 (1.46–85.36)	0.02	9.83 (1.22–79.27)	0.032
Mosaic architecture	12 (4.8)	3 (1.7)	2.91 (0.81–10.46)	0.102		
Fat-in-mass, more than adjacent liver	19 (7.6)	5 (2.8)	2.81 (1.03–7.68)	0.044		
Blood products in mass	18 (7.2)	3 (1.7)	4.48(1.30-15.43)	0.018		

Data (HCC and non-HCC columns) are the number of observations with the percentage in parentheses. Data in parentheses (OR columns) are 95% confidence intervals. ^a^Data refer to 65 HCCs and 69 non-HCC lesions that were examined by EOB-MRI, CE-MRI or CECT within the previous 6 months for which threshold growth could be measured; ^b^Data refer to 62 HCCs and 30 non-HCC lesions that were examined by EOB-MRI, CE-MRI or CECT within the previous 1 or 2 years for which the subthreshold growth could be measured. *HCC *Hepatocellular carcinoma, *OR *Odds ratio, *APHE *Arterial-phase hyperenhancement, *TP *Transitional phase, *HBP *Hepatobiliary phase

### Observation size with and without threshold growth

For all three lesion subgroups (HCC, non-HCC malignancy, and benign lesions), the mean sizes of the observed masses with threshold growth were smaller than those of the masses without threshold growth (14.8 vs. 22.6 mm, 22.1 vs. 32.6 mm, 9.7 vs. 17.8 mm, all *p* < 0.05). The comparison of observation size with and without threshold growth is shown in Table 4.

**Table 4:** Comparison of observation size with and without threshold growth

Observation	With threshold growth (mm)			Without threshold growth (mm)			*p* value
	Number	Mean size	Range	Number	Mean size	Range	
HCCs (*n* = 65)	32	14.8 ± 10.4	5–49	33	22.6 ± 10.8	10–55	0.003
Non-HCC malignancies (*n* = 37)	16	22.1 ± 16.7	5–70	21	32.6 ± 12.5	15–61	0.036
Benign lesions (*n* = 32)	9	9.7 ± 4.0	5–19	23	17.8 ± 9.9	8–52	0.026

*HCC *Hepatocellular carcinoma

### Diagnostic performance

Since the nodule-in-nodule architecture was an independently significant AF of HCC, in scheme B, threshold growth was replaced by this AF. According to the LI-RADS v2018, 35 lesions were LR-4, and 186 were LR-5. Of these, one HCC was downgraded from LR-5 to LR-4 based on scheme A, and 4 HCCs were upgraded from LR-4 to LR-5 based on scheme B. The results of the categorization of these observations are summarized in Table 5.

**Table 5:** Categories of observations

Category	LI-RADS v2018				Scheme A				Scheme B			
	HCC	Non-HCC malignancy	Benign lesion	Total	HCC	Non-HCC malignancy	Benign lesion	Total	HCC	Non-HCC malignancy	Benign lesion	Total
LR-1	0	0	20	20	0	0	20	20	0	0	20	20
LR-2	3	2	11	16	3	2	11	16	3	2	11	16
LR-3	18	2	28	48	18	2	28	48	18	2	28	48
LR-4	35	5	16	56	36	5	16	57	32	5	16	53
LR-5	186	11	9	206	185	11	9	205	189	11	9	209
LR-M	8	68	4	80	8	68	4	80	8	68	4	80
Total	250	88	88	426	250	88	88	426	250	88	88	426

Scheme A: categories assigned using all MFs except threshold growth, which was treated as an AF favouring malignancy, not HCC in particular, while the other algorithms were consistent with those of the LI-RADS v2018; Scheme B, nodule-in-nodule architecture replaced threshold growth as a new MF, while the other algorithms were consistent with scheme A. *HCC *Hepatocellular carcinoma

The sensitivities of schemes A and B were 74.0% and 75.6%, respectively, while the specificities were the same (88.6%). The metrics of the diagnostic performance for HCC (sensitivity, specificity, accuracy) of neither scheme A nor B were significantly different from those of the LI-RADS v2018 (all *p* > 0.05). A summary of the diagnostic performance of the LI-RADS protocols is shown in Table 6.

**Table 6:** Diagnostic performance of LI-RADS protocols for HCC

	Sensitivity	Specificity	PPV	NPV	Accuracy	Youden
LI-RADS v2018	74.4%	88.6%	90.3%	70.9%	80.3%	0.630
Scheme A	74.0%	88.6%	90.2%	70.6%	80.0%	0.540
Scheme B	75.6%	88.6%	90.4%	71.9%	81.0%	0.642
*P*_*a*_ value	> 0.999	> 0.999	—	—	> 0.999	—
*P*_*b*_ value	0.375	> 0.999	—	—	0.375	—

Schemes A and B are described in Table 5. *P*_a_ values are for comparisons of diagnostic performance between scheme A and LI-RADS v2018 using *McNemar’s* test, and*P*_b_ values are for comparisons of diagnostic performance between scheme B and LI-RADS v2018 using *McNemar’s* test. *PPV*, positive predictive value; *NPV*, negative predictive value

Schemes A and B are described in Table 5. *P*_*a*_ values are for comparisons of diagnostic performance between scheme A and LI-RADS v2018 using *McNemar’s* test, and *P*_*b*_ values are for comparisons of diagnostic performance between scheme B and LI-RADS v2018 using *McNemar’s* test. *PPV*, positive predictive value; *NPV*, negative predictive value.

## Discussion

In this study, according to univariate logistic regression analyses, threshold growth was not a specific feature for HCC diagnosis. Regardless of whether threshold growth was removed from the list of MFs or replaced by a nodule-in-nodule architecture (the independently significant AF for HCC), the sensitivity and specificity of the new LI-RADS algorithms for HCC were not significantly different from those of LI-RADS v2018.

Among the MFs, the APHE, washout and enhancing capsule were independently significant features for HCC diagnosis. APHE and washout showed high sensitivity for later-stage HCC as the arterial blood supply increased and the portal supply decreased in HCC [9, 10]. The specificity of washout was higher because it reflects the pseudocapsule composed of compressed fibrous tissue and dilated sinusoids around the HCC [11, 12]. The above three MFs have a definite pathological basis and are recognized by many HCC diagnosis and treatment guidelines [13, 14]. However, there is no strong pathological support for threshold growth in HCC. In a previous multivariable analysis of the LI-RADS, all MFs, except threshold growth (OR, 1.6; 95% CI, 0.7–3.6; *P* = 0.07), were associated with HCC [15]. In general, malignant tumours tend to grow faster and more easily show threshold growth, regardless of whether they are HCC. It has been reported that the tumour doubling times of HCC and intrahepatic cholangiocarcinoma (ICC) overlap [16, 17]. Because the features of some high-grade dysplastic nodules (DNs) are similar to those of early HCC [18], threshold growth also sometimes occurs in DNs. In this study, 7 DNs demonstrated threshold growth.

According to the definition of threshold growth (size increase of a mass by ≥ 50% in ≤ 6 months), when the lesion is small, it achieves threshold growth standard more easily. It has been previously reported that smaller HCCs demonstrate shorter doubling times [16, 19, 20]. In this study, all lesions (HCC, non-HCC malignancy, or benign lesions) demonstrating threshold growth were smaller in size than those without threshold growth (all *p* < 0.05). This finding further confirms the above conclusion.

Previous studies have shown that some special AFs contribute to the diagnosis of HCC [21, 22]. The nodule-in-nodule feature, a variant of the “mosaic” architecture of HCC, is a typical manifestation of tumour heterogeneity [23]. The development of most HCCs in patients with cirrhosis occurs via a multistep process of carcinogenesis, from a regenerative nodule (RN) to a DN and finally to HCC [24]. The components of different stages are prone to coexist in one nodule. Therefore, in the LI-RADS v2018, the nodule-in-nodule feature is an AF favouring HCC in particular. In our study, multivariate logistic regression analyses showed that a nodule-in-nodule appearance was an independently significant feature for HCC (*p* = 0.032) and was therefore selected to replace the threshold growth.

Chernyak et al. [5] reported that when threshold growth is removed, 9% of LR-5 observations will be downgraded to LR-4, which will affect the management of fast-growing LR-5 lesions and cause unnecessary biopsy. However, the aforementioned study was based on the LI-RADS v2014, and the concept of threshold growth at that time also included two other items, namely, new observations measuring ≥ 10 mm at two years and those that show a ≥ 100% diameter increase at > 6 months. Therefore, the proportion of lesions with threshold growth was as high as 66.4% in that report. Obviously, these data cannot represent the actual situation after the simplification of the threshold growth definition in the LI-RADS v2018, and the relatively large number of patients with prior exams may have inflated the importance of threshold growth. In addition, the article emphasizes only that removing threshold growth will reduce the number of LR-5 classifications but does not verify the diagnostic performance therein and cannot explain whether there are other non-HCC lesions misclassified as LR-5 due to threshold growth.

Another study reported that threshold growth was not a significant diagnostic indicator of HCC and was more common in non-HCC malignancies [6]. The conclusion of that paper was similar to that of our study. In addition, unlike other MFs, threshold growth cannot be measured for all observations; specifically, the number of observations for which threshold growth can be measured should be far lower than the total number of observations. In our study, the percentage of lesions that had been subjected to prior examination and measurements of threshold growth was only 31.5% (134/426); this percentage is lower than the 43.2% reported by Park et al. [6]. It is also worth noting that for certain patients with lesions in our institution, threshold growth could not be evaluated because they were actively treated when the lesions were first discovered and therefore did not meet the criterion requiring no treatment between the two examinations. In the real world, the number of lesions for which threshold growth can be evaluated is relatively small, which is also one of the reasons for its limited role in HCC diagnosis.

This study had several limitations: First, there may have been selection bias due to the retrospective study design, the inclusion of hepatic observations confirmed by histopathology and the exclusion of HCC confirmed by typical imaging findings. Second, few LR-1 and LR-2 nodules were included in this study due to the lack of pathological diagnosis. Third, the sample size of patients with observations with threshold growth was small because few patients avoided treatment between the two examinations.

In conclusion, threshold growth is not an independently significant MF for HCC diagnosis. The diagnostic performance of the LI-RADS for HCC is not affected regardless of whether threshold growth is removed from the MFs or replaced by an independently significant AF, which is the nodule-in-nodule feature. In other words, threshold growth has a limited role in differentiating HCC from other focal hepatic lesions.

## Data Availability

Not applicable.

## Abbreviations

LI-RADS: Liver Imaging Reporting and Data System
LI-RADS v2018: LI-RADS version 2018
ACR: American College of Radiology
OPTN: Organ Procurement and Transplantation Network
MFs: Major features
AFs: Ancillary features
HCC: Hepatocellular carcinoma
CT: Computed tomography
CECT: Contrast-enhanced CT
MRI: Magnetic resonance imaging
EOB-MRI: Gadoxetic acid–enhanced MRI
CE-MRI: Contrast-enhanced MRI
DWI: Diffusion-weighted imaging
GRE: Gradient recalled echo
TSE: Turbo spin echo
VIBE: Volumetric interpolated breath-hold examination
TR: Repetition time
TE: Echo time
FA: Flip angle
ST: Slice thickness
SS: Slice spacing
FOV: Field of view
T1WI: T1-weighted imaging
T2WI: T2-weighted imaging
APHE: Arterial-phase hyperenhancement
TIV: Tumour-in-vein
NASH: Nonalcoholic steatohepatitis
ICC: Intrahepatic cholangiocarcinoma
CHC: Combined hepatocellular cholangiocarcinoma
DN: Dysplastic nodule
RN: Regenerative nodule
FNH: Focal nodular hyperplasia
AP: Arterial phase
PVP: Portal venous phase
TP: Transitional phase
HBP: Hepatobiliary phase
DP: Delayed phase
PPV: Positive predictive value
NPV: Negative predictive value
